# Application of Low-Temperature Air Plasma for the Enhancement of Defense Fabric’s Self-Cleaning Property

**DOI:** 10.1021/acsomega.4c06893

**Published:** 2024-09-20

**Authors:** Shilpi Akter, Md. Faisal Mahmud, A. N. M. Masudur Rahman, Nadvi Mamun Pritha, Md. Mahmudul Hasan, Md. Hedayet Ullah, Md. Rowshanuzzaman Kanon, Fayeeka Tasnim Ahona, Bebe Fatema Bristy

**Affiliations:** † Department of Fabric Engineering, Faculty of Textile Engineering, Bangladesh University of Textiles (BUTEX), Dhaka 1208, Bangladesh; ‡ Department of Materials, Faculty of Science & Engineering, University of Manchester, Manchester M13 9PL, U.K.; § Department of Textile Engineering, Ahsanullah University of Science and Technology, Dhaka 1208, Bangladesh; ∥ Key Laboratory of Textile Science & Technology, Ministry of Education, College of Textiles, Donghua University, Shanghai 201620, China; ⊥ Department of Textile Engineering, Primeasia University, Dhaka 1213, Bangladesh; # Department of Apparel Engineering, Bangladesh University of Textiles (BUTEX), Dhaka 1208, Bangladesh; ¶ Department of Physics, Bangladesh University of Textiles (BUTEX), Dhaka 1208, Bangladesh; ∇ Department of Dyes and Chemical Engineering, Bangladesh University of Textiles (BUTEX), Dhaka 1208, Bangladesh

## Abstract

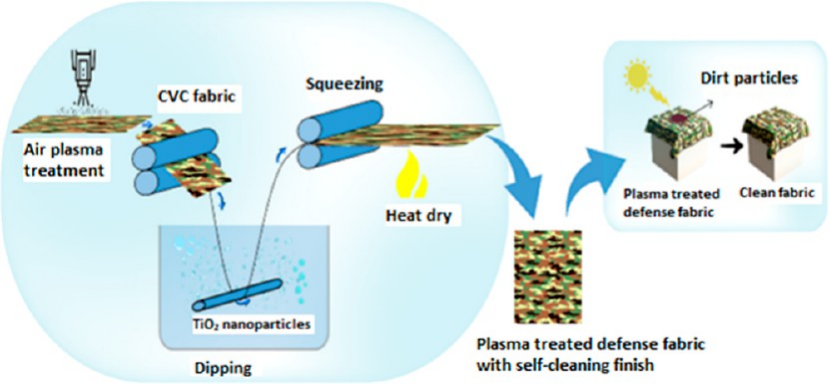

Self-cleaning textiles have the potential to revolutionize the lives of people like military personnel and hikers who spend extended periods of time in the sun and have restricted access to washing facilities. This research aims to develop the self-cleaning capability of defense uniforms by utilizing air plasma treatment and applying TiO_2_ nanocoating. Following plasma treatment of differing durations (2, 4, 6, 8, and 10 min, respectively), a pad-dry cure method was employed to apply a TiO_2_ coating to each sample, while keeping other processing parameters constant. SEM, Fourier transform infrared spectroscopy, ultraviolet protection factor (UVPF), energy-dispersive X-rays, and a water contact angle test were performed in order to validate the air plasma-induced surface modification. There was a gradual escalation in the rate of TiO_2_ absorption with an extension of the plasma treatment duration. Afterward, the samples were stained with various organic and inorganic compounds, including oil, ink, soil, and coffee, and subsequently exposed to sunlight for a period of 6 h. The samples demonstrated an enhanced cleaning effectiveness with increasing quantities of TiO_2_. The reflectance value and visual assessment of washed sample showed a reduced yet still present self-cleaning characteristic. The UVPF of the samples increased gradually as the duration of plasma treatment increased due to the UV absorption properties of TiO_2_, as validated by measuring the band gap energy.

## 1 Introduction

The demand for functional fabrics has experienced a significant surge in the dynamic contemporary world, leading to numerous exhilarating advancements within the textile industry. Self-cleaning fabric, also known as biomimetic fabric, is inspired by the remarkable “lotus effect”.^1^ In addition to lotus leaves, other materials such as rice leaves,^2^ butterfly wings, salvinia molesta,^3^ shark skin,^4^ fish scales, and mosquito eyes^5^ also exhibit self-cleaning property.

**Figure 1:**
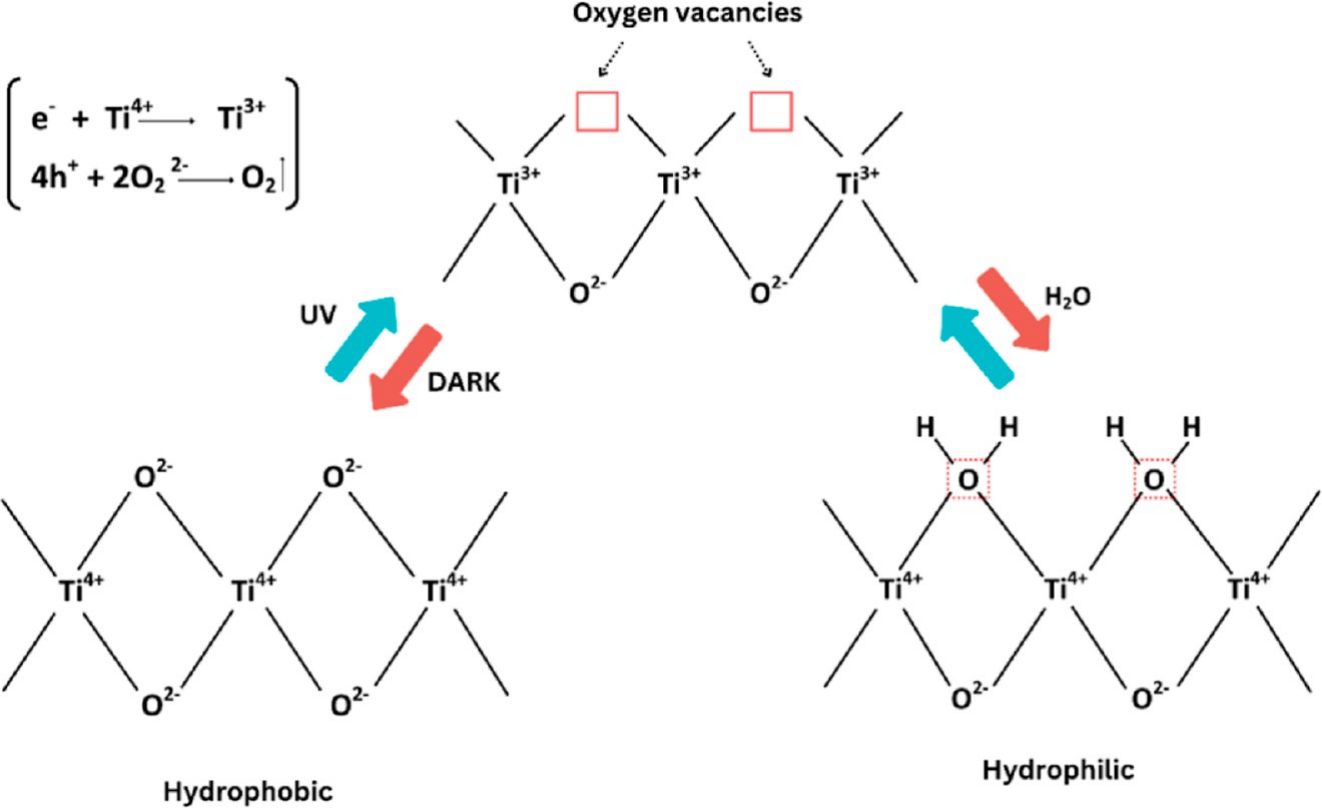
Photo activity of TiO_2_. Adapted with permission from ref (15), Copyright 2020 Elsevier.

In recent years, the textile industry has experienced a substantial growth in the implementation of nanoparticles (diameter ranging from 1 to 100 nm) due to their remarkable capacity to enhance the longevity and functionality of fabrics.^6,7^ Among the nanoparticles, Al_2_O_3_, ZnO, and TiO_2_ exhibit commendable self-cleaning characteristics.^8^ The profitability of employing TiO_2_ in textiles is attributed to its availability, nontoxic nature, affordability, and chemical stability. Furthermore, the application of nano TiO_2_ coating on materials does not affect their breathability or tactile sensation.^9^ Wang et al. reported the discovery of photoinduced super hydrophilic behavior in TiO_2_ and categorized it as a photoresponsive wetting agent capable of degrading synthetic dyes and organic contaminants.^10^ TiO_2_ exhibits light absorption when subjected to energy sources with a higher magnitude than its band gap, which is 3.2 eV.^11^ Consequently, the excited electrons transition to the conduction band, whereas the hole remains in the valence band. The generated electrons (e^–^) and holes (h^+^) can either recombine and emit the excess energy as heat or light, or they can recombine without emitting radiation.^12^ The hole (h^+^) ions in the valence bonds react with water to generate hydroxyl radicals, while the electrons in the conducting band transform surface oxygen into superoxide radicals. The resulting product of the combination of these superoxide radicals and water is a hydroxyl radical. The reactive oxygen species generated through free-radical processes decompose the volatile organic molecules into CO_2_ and H_2_O.^13^ The photocatalytic activity has the ability to break down organic substances, such as odorants, viruses, and germs present in the air.^14^ The main processes involved were the reconstruction of the Ti–OH link triggered by light and the formation of oxygen vacancies. Figure 1 illustrates the mechanism.

Qi et al. demonstrated that cotton fabric treated with TiO_2_ nanoparticles using the sol–gel process exhibits a self-cleaning characteristic.^16^ The study conducted by Galkina et al. demonstrated that the incorporation of a cross-linking agent onto the cotton surface results in improved self-cleaning capability and increased durability of nanoparticles on the fabric.^17^ In their study, Kathirvelu et al. discovered that fabric composed of a blend of cotton and polyester, which underwent nanoparticle treatment, had superior stain removal capabilities compared to cotton fabric.^18^ Nevertheless, surface modification is necessary to guarantee the stability of nanoparticles on the fabric surface. By applying chemical pretreatment to wool-polyester blended fabric coated with TiO_2_, Montazer and Seifollahzadeh established that the fabric’s capacity for self-cleaning is enhanced. Nevertheless, the fabric that underwent chemical pretreatment did not attain a significant level of cleaning effectiveness.^19^ Physical surface modification techniques can alter the fabric’s absorbency without affecting its internal characteristics. As a method for altering the reactivity of polymer surfaces, plasma treatment is remarkably effective.^20^ The procedure elicits the development of polar substituents onto the fabric surface, facilitating a robust connection between nanoparticles and fabric surfaces.^21^

Rabiei et al. tested the ultraviolet protection factor (UVPF) of cotton-polyester twill fabrics (70–30%) covered with TiO_2_ nanoparticles synthesized in situ. The findings revealed that the fabric coated with TiO_2_ nanoparticles had higher UVPF values than the uncoated textile, while the inherent qualities of the coated fabric did not change considerably. Based on the findings, it is possible to conclude that coating work-wear fabrics with TiO_2_ nanoparticles improves their UV protection without affecting the cooling impact of perspiration evaporation.^22^ Dumitrescu et al. researched how to make long-lasting self-cleaning textiles by coating fabrics with TiO_2_–(1%) Fe–N–graphene (2%). The textiles coated with doped TiO_2_-graphene particles are particularly hydrophilic because a large number of hydrophilic TiO_2_ particles are deposited on the material’s surface. The coated cotton/polyester fabrics with graphene oxide/TiO_2_ nanocomposite have good photocatalytic self-cleaning activity as measured by the degradation of methylene blue under visible light irradiation.^23^ Abualnaja et al. proposed a successful method for designing and preparing Ag/TiO_2_ nanocomposite particles capable of imparting high antimicrobial, photocatalytic, and self-cleaning activities to plasma-activated fabrics under ultraviolet and visible light, making them potentially useful as multiple functional materials for a variety of applications.^24^ However, there is a lack of research examining the potential effects of plasma surface modification on defense fabrics, in terms of improving their self-cleaning properties.

This study primarily focuses on the utilization of plasma treatment on cotton-polyester blended fabric with the objective of creating a self-cleaning characteristic specifically designed for military people. The main aim is to develop a surface that is capable of cleaning itself, minimizing the amount of time required for cleaning, streamlining the maintenance process, and protecting the environment through the exploration of an efficient approach. The plasma treatment is applied to the surface of a CVC woven fabric, followed by the application of self-cleaning nanoparticles. To accomplish this, sections of cloth were exposed to plasma treatment for durations of 2, 4, 6, 8, and 10 min prior to being infused with 1% TiO_2_ nanoparticles. The untreated and plasma-treated samples underwent analysis utilizing Fourier transform infrared spectroscopy (FTIR), scanning electron microscopy (SEM), and energy-dispersive X-ray (EDX), a self-cleaning test, wettability, color fastness to rubbing, tensile strength, and air permeability. Soldiers involved in warfare or training sometimes face harsh conditions, where the supply of water is usually insufficient. Consequently, there is a strong demand for an advanced fabric that possesses self-cleaning properties, specifically designed for the use of military people.

## 2 Experimental Methods

### 2.1 Materials

The study involved the collection of camouflage printed CVC fabric from Rahim Textile Ltd. in Bangladesh. The fabric composition is verified using a suitable method for composition testing. The fabric’s analyzed specification is provided in Table 1. To prevent contamination, the samples were cleaned by using a detergent solution with a concentration of 1.5 g/L. Subsequently, the fabric underwent a treatment using anatase TiO_2_ (10–25 nm, Nuco Chemicals, China). The particle size was confirmed using a particle size analyzer (FRITSCH, Germany).

**Table 1:** Fabric Specifications

parameters	value
fabric type	CVC (70% cotton, 30% polyester)
fabric structure	3/1 Z twill
yarn linear density (Ne)	warp: 22, weft: 24
yarns per cm	warp: 49, weft: 29
GSM (gram per square meter)	320

### 2.2 Plasma Treatment

**Figure 2:**
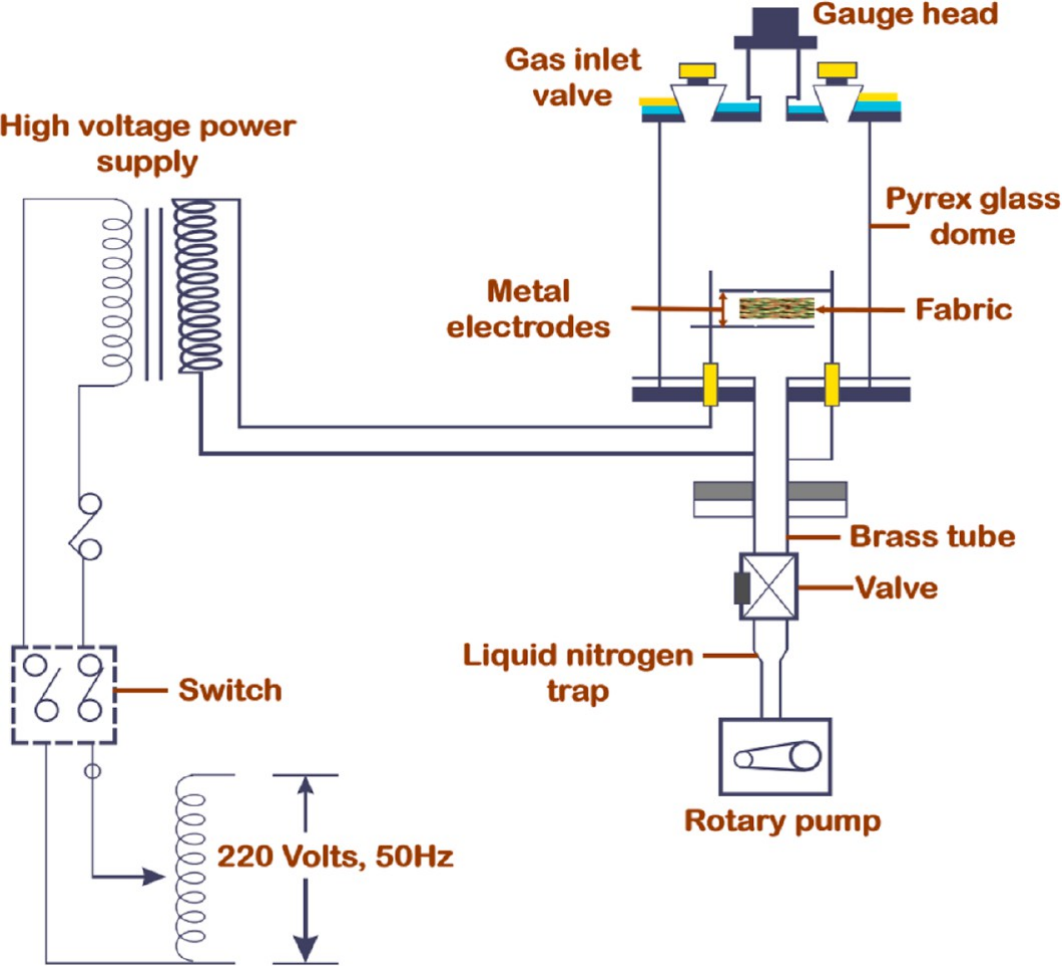
Schematic illustration of the plasma treatment chamber.

Plasma treatment is an environmentally friendly technique that is utilized to improve the surface properties of a fabric. The printed fabric undergoes plasma treatment by using an air plasma system, which yields optimal outcomes. Figure 2 depicts a process diagram of the air plasma chamber. The fabric was positioned within a plasma chamber enclosed by a pyrex glass bell jar. The jar measured 15 cm in diameter and 18 cm in length. Within the plasma chamber, two circular electrodes measuring 1 cm in thickness and 9 cm in diameter were positioned at a distance of 4 cm. The plasma treatment specimen was positioned on the lower electrode. After placing the sample into the chamber, a rotary pump was employed to facilitate its evacuation. It regulates the pressure during plasma treatment. Printed fabrics underwent plasma treatment in a glow discharge plasma chamber that was capacitively coupled to an alternating current (AC) source. In this study, a plasma power supply (model: HPT-200, Henniker Plasma, Henniker Scientific Ltd., UK) delivering 200 W of power at a frequency of 40 kHz was used for treating the surfaces of the samples. The efficacy of plasma treatment is contingent upon factors such as the duration,^25^ magnitude,^26^ electrical parameters, and gas conditions.^27^ The experiment involved conducting plasma treatment at time intervals of 2, 4, 6, 8, and 10 min, using a pressure of 0.2 mbar with a flow rate of 6 cm^3^/s. Typically, there are two categories of plasma systems employed for altering surfaces: cold plasma and hot plasma. Low-pressure glow discharge plasma represents a form of cold plasma. The chamber temperature typically ranges between 40 and 50 °C during treatment, ensuring that samples remain unburned when plasma is generated.

In this work, air was used as a treatment gas. Initially air is introduced into the plasma chamber at a low pressure of 0.2 mbar. Then, an AC electric field is applied to the gas, leading to the ionization of gas molecules. This results in the formation of a plasma that consists of highly energized ions, electrons, free radicals, and other reactive species. Reactive species in plasma chemically react with the surface molecules of the sample. As a result, oxygen-containing functional groups are formed and increase the surface energy and wettability.

### 2.3 Pad–Dry–Cure Method

The sample was subjected to the pad–dry–cure process, where it was coated with nano TiO_2_. The samples underwent treatment with a 1% concentration of TiO_2_ at a liquor ratio of 1:50 for a duration of 10 min. At first required amount of water and nano TiO_2_ were taken into a beaker and stirred for 5 min to ensure proper mixing with water, and then, the sample was continuously stirred to guarantee the impregnation of TiO_2_ on the fabric surface. Following the treatment, the samples were processed using a sample padder machine, resulting in a 75% pick-up. The samples were subsequently dehydrated in a dryer for a duration of 5 min at a temperature of 100 °C and followed by curing 3 min at 120 °C in an oven.

### 2.4 Physical and Mechanical Properties

The fabric’s physical qualities, such as air permeability, color fastness to rubbing, and GSM, as well as mechanical properties, such as tensile strength, have been investigated. Prior to testing, all fabric specimens underwent conditioning at (65 ± 2)% RH and (27 ± 2) °C for a duration of 24 h, in accordance with ASTM D1776 and BS EN 2013. The outcome was determined by averaging the values of five samples. The air permeability test of the sample was carried out at both state by following ASTMD 737 standard with the using air permeability tester (Model FX-3300). The air permeability of fabrics was statistically evaluated using a one-way analysis of variance (ANOVA) executed using SPSS v.20 software. The statistical significance of the variance was determined *p* < 0.05 level. The assessment of color fastness to rubbing was conducted using the ISO 105 X12:2002 method. The tensile strength of fabrics was assessed using the ASTM D5035-11:2015 strip method at a speed of 300 mm/min, employing the Universal strength tester.

### 2.5 Fourier Transform Infrared (FTIR) Spectroscopy

The sample underwent analysis of FTIR spectroscopy, utilizing PerkinElmer equipment within the frequency range of 500–4000 cm^–1^. Typically, two scans were performed for each sample.

### 2.6 Band Gap Energy

To interpret the photocatalytic activity, it is crucial to know the band gap energy of TiO_2_. The spectrum of TiO_2_ in the particle form was measured using a Cary 60 UV–vis spectrophotometer (Agilent Technology, USA). The wavelength spanned from 250 to 800 nm. The machine was operated in a dual mode, scanning at a rate of 600 nm/min, with a data interval of 1 nm.

### 2.7 Ultraviolet Protection Factor (UVPF)

The measurement of UVPF is typically conducted to assess the fabric’s ability to effective protection against harmful UV radiation. The UVPF was determined in accordance with EN 13758-1:2001 standard by evaluating the UV transmission spectra (290–400 nm) at 5 nm intervals using the Cary 5000 UV–vis–NIR spectrophotometer (Agilent Technology, USA).

### 2.8 Scanning Electron Microscopy (SEM)

SEM was employed to analyze alterations in the morphology of a specimen. The untreated and nanoparticle-treated samples were coated with gold and analyzed using FE-SEM equipment (ZEISS, Germany) with a 10 kV accelerating voltage.

### 2.9 Water Contact Angle

The AATCC 79-2000 procedure was employed to determine the contact angle formed by the interface of water and the fabric surface.^28^ Each droplet was captured instantaneously using a Canon EOS 600D camera, and the contact angle was measured using Photoshop software.^29^ This methodology was employed to investigate the wetting properties of untreated fabric, fabric treated with only plasma, and TiO_2_-coated fabric treated with plasma.

### 2.10 Energy-Dispersive X-ray (EDX)

The atomic composition of the elements in the treated fabrics was determined by using an EDX device coupled to a scanning electron microscope to determine the proportion of their respective contents. An analysis was conducted to compare the quantity of metals found on the surface of plasma-treated and untreated fabrics.

### 2.11 Self-Cleaning Property

**Figure 3:**
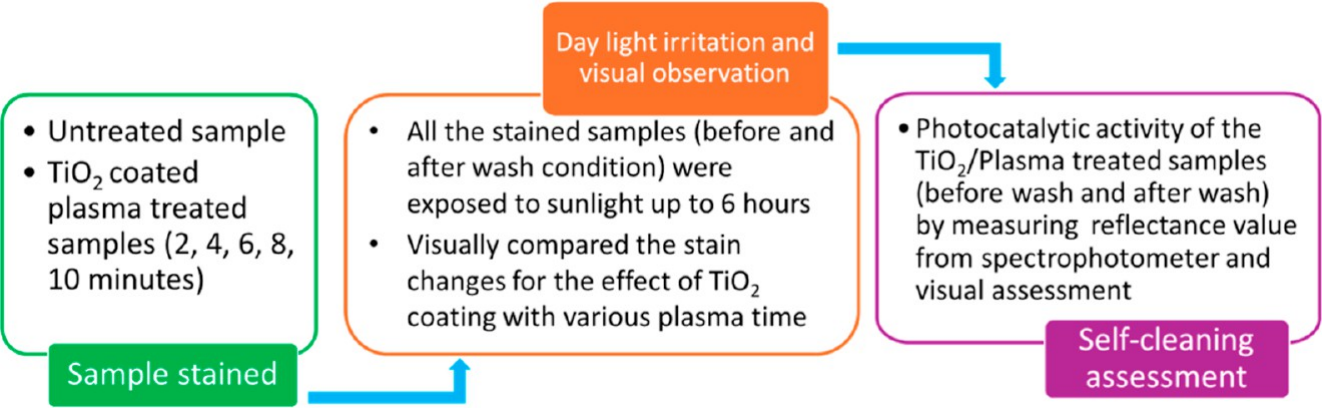
Self-cleaning property scheme of untreated and plasma-treated fabrics.

The fabric’s self-cleaning properties were assessed using day light irradiation.^28^ Both untreated and TiO_2_-coated plasma-treated cloth was subjected to the application of oil, ink, soil, and coffee stains. Upon exposing the stained sample to sunlight for a duration of 6 h, a noticeable change in the spot became apparent. A clear comparison has been demonstrated between untreated fabric and plasma-treated fabric by visual assessment and measuring reflectance value from a spectrophotometer (X-rite, USA) within the wavelength range of 360–760 nm. Figure 3 illustrates the self-cleaning assessment scheme.

### 2.12 Self-Cleaning Property after Wash

To determine the durability and stability of the TiO_2_ coating on the fabric surface, the samples were subjected to five cycle washing by detergent and dried under environmental conditions following the ISO-105-C01 method. After drying, the self-cleaning property of the washed samples was evaluated by measuring the reflectance value and conducting a visual inspection.^28^

## 3 Results and Discussion

### 3.1 Scanning Electron Microscopy

**Figure 4:**
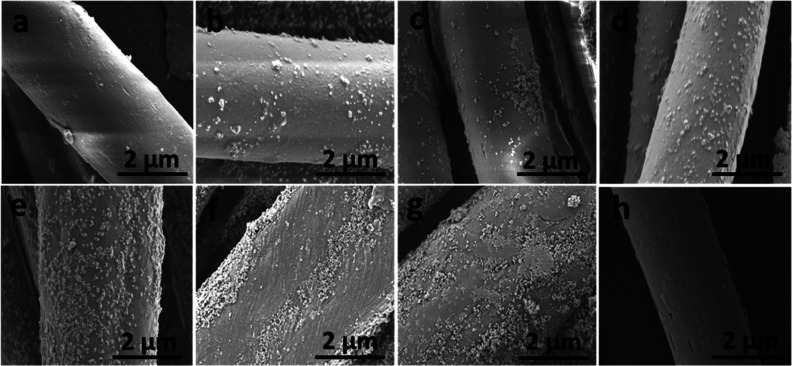
SEM analysis of (a) untreated, (b) TiO_2_ coated without plasma, (c) TiO_2_-coated 2 min plasma-treated, (d) TiO_2_-coated 4 min plasma-treated, (e) TiO_2_-coated 6 min plasma-treated, (f) TiO_2_-coated 8 min plasma-treated, (g) TiO_2_-coated 10 min plasma-treated fabrics, and (h) 10 min plasma treated without TiO_2_ coating.

The SEM images of CVC fabric samples, both untreated and treated with plasma, are displayed in Figure 4. The plasma treatment was conducted for different durations of 2, 4, 6, 8, and 10 min. The visual representations illustrate the modifications that occur on the surface of the fabric as a consequence of plasma treatment. Figure 4a illustrates the pristine surface of untreated fabric, which is remarkably smooth, whereas Figure 4b depicts the surface of TiO_2_-coated fabric in the absence of plasma treatment, where a negligible quantity of TiO_2_ has deposition on the fabric surface. Figure 4c–h illustrates that the fabric surface irregularity increases, and damage becomes discernible when the plasma treatment duration is increased from 2 to 10 min. The plasma’s elevated concentration of reactive ions enables it to interact with the fabric’s surface and disrupt chemical bonds.^30^ Plasma treatment has the ability to cause modifications in both physical and chemical compositions. Prolonged exposure of fabric to plasma results in the formation of cracks and an increase in the abrasiveness. Figure 4 demonstrates that the presence of fractures on the fabric surface is more pronounced in plasma-treated fabric as compared with untreated fabric. It is evident that a longer time of plasma treatment results in greater deposition of TiO_2_ on the surface. Several research studies have showed similar results by examining the effects of plasma treatment on fabric surfaces.^31^ At the beginning, the reactive ions engage with the amorphous section of the fabric surface, which is less tightly bound than the crystalline section. The main goal is to specifically remove the amorphous portion while maintaining the structural integrity of the crystalline portion.^32^ Over an extended period of treatment, chemical linkages are disrupted as the surface roughness increases. The surface qualities of fabric play a crucial role in promoting chemical reactions and improving the material’s capacity to absorb and distribute liquid efficiently.^29^

### 3.2 Energy-Dispersive X-ray Analysis

**Figure 5:**
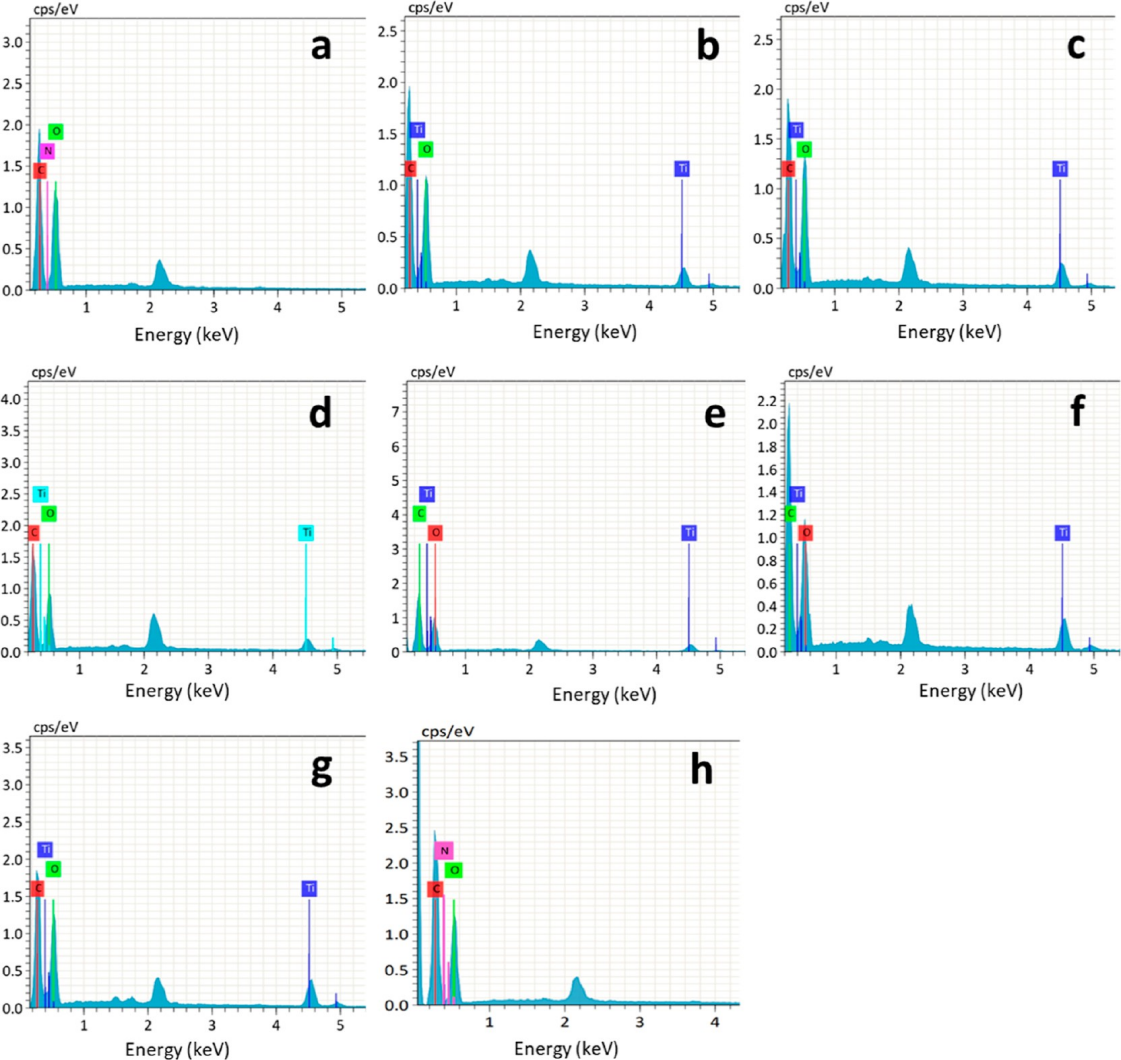
EDX analysis of (a) untreated fabric, (b) TiO_2_-coated fabric without plasma treatment, (c) TiO_2_-coated 2 min plasma-treated fabric, (d) TiO_2_-coated 4 min plasma-treated fabric, (e) TiO_2_-coated 6 min plasma-treated fabric, (f) TiO_2_-coated 8 min plasma-treated fabric, (g) TiO_2_-coated 10 min plasma-treated sample, and (h) 10 min plasma treated without TiO_2_ coating.

EDX is utilized to quantify the amount of titanium (Ti) present in various fabric samples. The results depicted in Figure 5a indicate that the untreated sample does not exhibit any discernible peak for Ti. The sample represented in Figure 5b is subjected to TiO_2_ treatment in the absence of plasma. This sample exhibits a diminished peak of Ti in comparison to other samples that underwent plasma treatment and were coated with nanoparticles. The peak of Ti in samples Figure 5c–g shows a progressive increase as the plasma treatment period increases. The extracted data have been organized in Table 2. The data presented in Table 2 demonstrate that an extended duration of plasma treatment results in an elevated level of chemical absorption on the surface of the fabric, leading to an increased level of Ti deposition on the surface of the cloth. The SEM images also verify this increase.

**Table 2:** EDX Test Results

sample	C (%)	O (%)	N (%)	Ti (%)	total (%)
untreated fabric	45.11	47.41	7.48		100
TiO_2_-coated fabric without plasma treatment	44.44	40.75		14.81	100
TiO_2_-coated 2 min plasma-treated	40.58	42.85		16.58	100
TiO_2_-coated 4 min plasma-treated	43.63	39.77		16.60	100
TiO_2_-coated 6 min plasma-treated	43.44	39.31		17.25	100
TiO_2_-coated 8 min plasma-treated	42.17	38.38		19.45	100
TiO_2_-coated 10 min plasma-treated	37.86	39.66		22.48	100
10 min plasma treated without TiO_2_ coating	40.28	44.14	15.58		100

### 3.3 Analysis of Wettability

**Figure 6:**
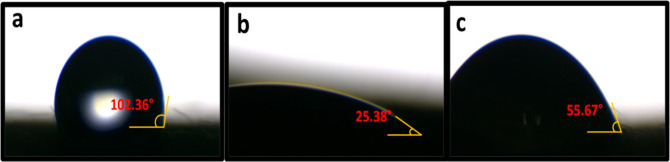
Water contact angle of CVC fabric (a) untreated, (b) sample with plasma treatment for 2 min without TiO_2_ coating, and (c) sample with plasma treatment for 2 min with TiO_2_ coating.

Textile material wettability can be determined by measuring the contact angle generated by a water droplet. The wettability calculation took into account only the side of the fabric that had been treated with plasma. In Figure 6a, the contact angle of an untreated fabric is measured to be 102.36°. Surface plasma treatment enhances the fabric’s polarity by reducing the contact angle between water and the fabric.^33^ Following a 2 min exposure to plasma without TiO_2_ coating, the fabric’s contact angle with a water droplet fell down to approximately 25.38°, as illustrated in Figure 6b. The cloth’s hydrophilicity is inversely proportional to its contact angle.^34^ Nevertheless, the contact angle of the plasma treated and impregnated with TiO_2_ is approximately 30° greater than that of the fabric without TiO_2_ impregnation, as depicted in Figure 6c. The augmented contact angle is attributed to the existence of a TiO_2_ coating on the cloth. Extended application of plasma increases the number of reactive species such as hydroxyl and carboxylic attached to the fabric surface during the plasma oxidation method, which increased the wicking ability of the cloth. As a result, capillary pressure is reduced by increasing the fabric’s surface roughness.^29,35,36^

### 3.4 FTIR Analysis

**Figure 7:**
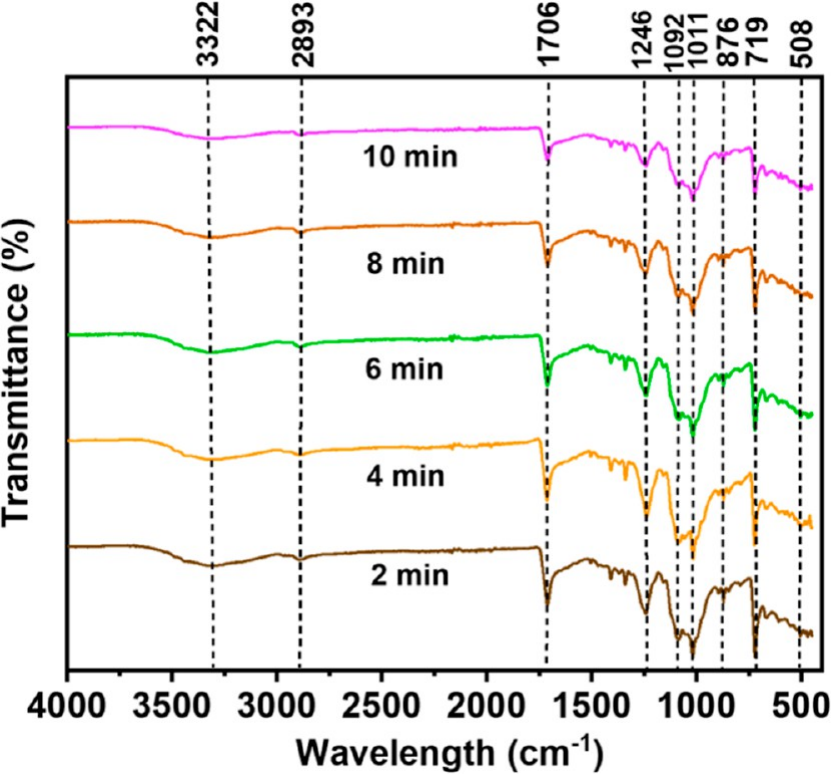
FTIR spectra of TiO_2_-coated plasma-treated fabrics at various times (2, 4, 6, 8, and 10 min).

Figure 7 depicts the FTIR spectra of the TiO_2_-coated plasma-treated samples with different times (2, 4, 6, 8, and 10 min). Transmittance has been used to study FTIR spectra. It is observed that the FTIR curves do not differ due to the time variation of the plasma treatment. The spectra exhibited enhanced transmission of OH groups in the wavelength range of 3000–3500 cm^–1^ as a result of the addition of hydroxyl groups through plasma treatment. The stretching vibrations of C–H and C=O are represented by the peaks at 2893 and 1706 cm^–1^, respectively. These results demonstrate the role of plasma treatment in the enhancement of functional groups. The plasma treatment removes radicals from the outer surface of the fabric and disrupts the carbon chain in cellulose by introducing various functional groups. The reaction between anionic, cationic radicals of O, and atomic groups of O results in the formation of groups such as CH_2_–OH, C=O, −COOH, and −COH. The findings also show that plasma treatment causes the formation of active groups on the surface. These active groups promote hydrophilicity and chemical absorption. The absorption bands at 508 and 719 cm^–1^ were induced by the bending vibrations of the Ti–O and the O–Ti–O groups, respectively, while the peak at 1092 cm^–1^ was due to the bending vibration of Ti–O–Ti. Thus, it may be inferred that the fabrics were effectively coated with TiO_2_ nanoparticles. These findings align with previous research conducted on TiO_2_ nanoparticles.^37−39^

Plasma treatments are used for activating, cleaning, and modifying various materials, including polymers. The goal of plasma treatment is to transform a low-energy surface into a high-energy surface by eliminating surface contaminants and bonding oxygen-containing molecules onto it. Plasma contains highly excited ions, free radicals, and reactive species, which exhibit intense reactivity with particles or surfaces with which they come into contact with. They are also highly energetic to break existing chemical bonds and formed new ones as evidenced by FTIR analysis.^28,37^

### 3.5 Analysis of Band Gap Energy

**Figure 8:**
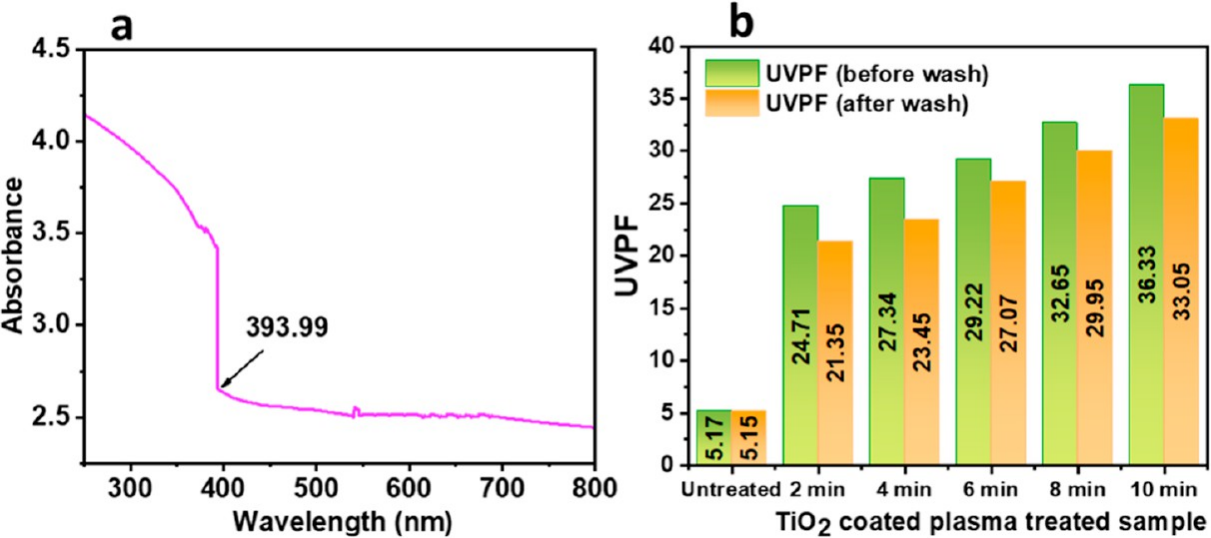
(a) UV–vis spectrum of the TiO_2_ nanoparticles and (b) UV protection factor values for untreated and TiO_2_-coated plasma-treated samples.

The UV–vis spectra of the TiO_2_ nanoparticles have been displayed in Figure 8a. The data were measured in the absorbance mode. The obtained spectrum shows a strong cut of 393.99 nm wavelength. Calculation of the energy band gap was performed by using the following formula^40^
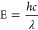
where wavelength, λ = 393.99 × 10^–9^ m, velocity of light, *c* = 3 × 10^8^ m/s, Plank constant, *h* = 6.626 × 10^–34^ J s, therefore, band gap energy, *E* = 5.05 × 10^–19^ e = 3.15 eV. The energy band gap of TiO_2_, specifically in its anatase form, guarantees its reactivity in the ultraviolet (UV) light range.^41^

### 3.6 UVPF Analysis

Figure 8b displays the calculated UVPF values of untreated and TiO_2_/plasma-coated fabrics. The UVPF was calculated using the following equation^42^
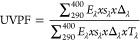
where *E*_λ_ = irradiation source efficiency W·m^–2^·nm^–1^, *S*_λ_ = erythemal effectiveness value from CIE, and Δ_λ_ = wavelength interval. From Figure 8b, it is evident that the untreated sample had a low UVPF value. However, by TiO_2_ coating through various plasma treatment durations, the UVPF values showed a gradual increase. As a result, sample treated with plasma for 10 min had the highest UVPF value. The increase in UVPF values is a result of the inherent UV absorption characteristics of TiO_2_, as elucidated by the band theory of solids.^22,43^ TiO_2_ is a type of semiconductor, possesses a broad band gap of (3.15 eV) between the low-energy valence and high-energy conduction bands.^44^ Activating TiO_2_ with light waves over its band gap causes electrons to absorb UV light, resulting in UV radiation protection. The UVPF rating of the postwash sample is lower in comparison to the prewash samples. While washing, the quantity of TiO_2_ decreased, resulting in a lower UVPF value. The findings are consistent with previous research.^45^

### 3.7 Assessment of Self-Cleaning Property

**Figure 9:**
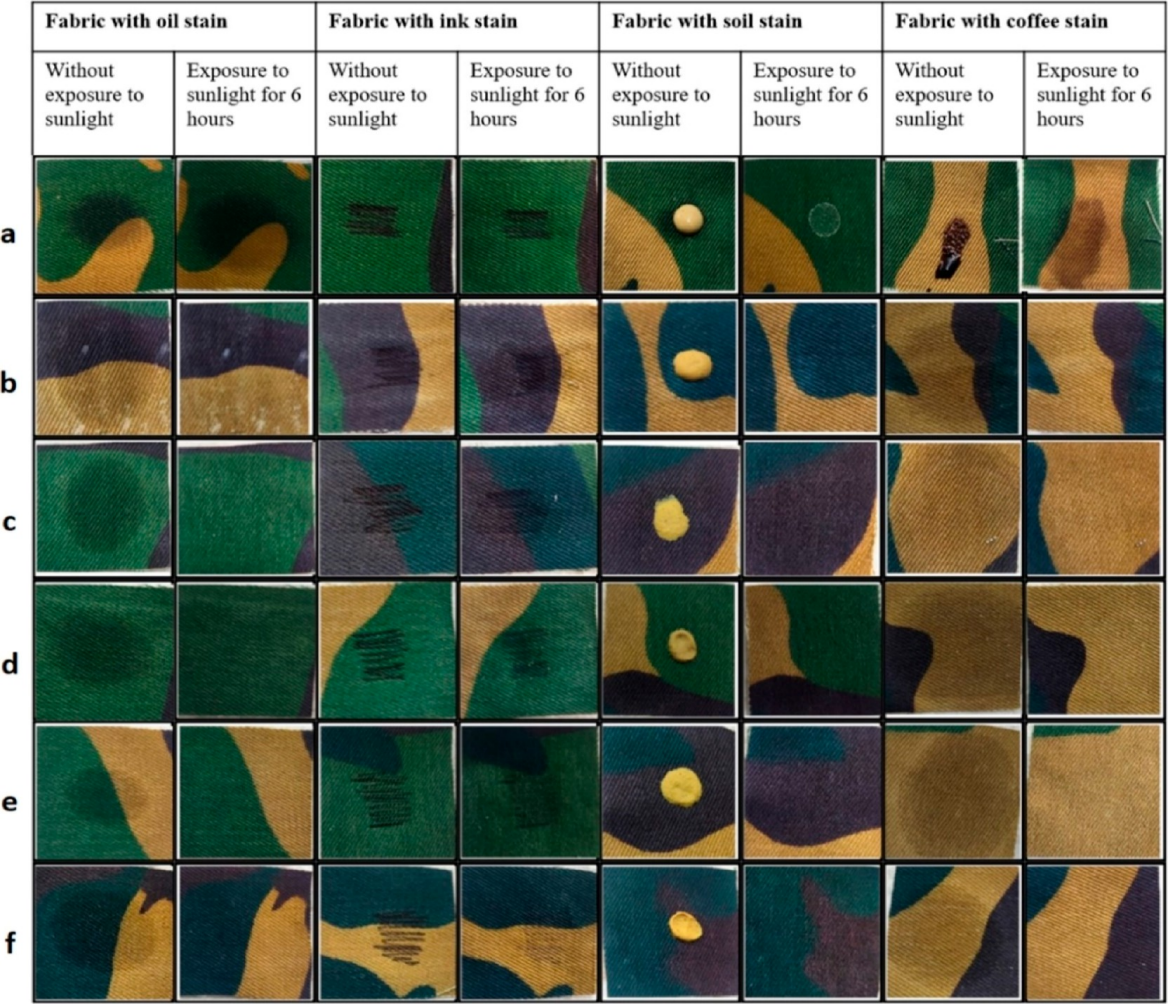
(a) Untreated sample, (b) TiO_2_-coated sample treated with plasma for 2 min, (c) TiO_2_-coated sample treated with plasma for 4 min, (d) TiO_2_-coated sample treated with plasma for 6 min, (e) TiO_2_-coated sample treated with plasma for 8 min, and (f) TiO_2_-coated sample treated with plasma for 10 min.

The nanosized anatase form of TiO_2_ exhibits the characteristic of self-cleaning. The primary rationale for using this material is its affordability and lack of toxicity. TiO_2_ exhibits photocatalytic properties. This means that when exposed to sunlight/UV light in the presence of water, it generates oxygen radicals. These oxygen radicals have the ability to break down organic and inorganic substances, including fats, oils, and plant materials. Titanium dioxide has high reactivity when it is in nanoform. It does not expend during catalysis; hence, the impact is long-lasting. On these self-cleaning surfaces, organic dirt is dissolved in the water, degraded, and then eliminated by the subsequent wash.^28^Figure 9 illustrates the results of untreated fabric and fabric treated with nanocoating and plasma treatment over different time intervals for stains caused by oil, ink, oil, and coffee after a 6 h exposure to light.

Upon applying oil to the fabric surface, it was found that after a 6 h exposure to light, sample (a) exhibited an oil stain. However, samples (b–f) do not exhibit any observable traces of oil. The hydrocarbon chain in oil is broken down by reactive oxygen species, resulting in their assault.^13,46^ The application of ink yields distinct outcomes compared to those of the application of oil strain. Sample (a) exhibited no discernible change in the ink spot after being illuminated for 6 h. However, samples (b–f) exhibit a noticeable disparity in the ink spot appearance upon exposure to UV light. The sample (f) exhibits the most effective self-cleaning outcome compared to the other samples. However, a discernible scene can still be demonstrated in sample (f). The samples with lower treatment time showed less self-cleaning efficiency in terms of ink as ink comprises dyes, pigment, and other chemicals with intricate composition that is resistant to degradation by the photolytic effect of TiO_2_.^47^ However, when the plasma treatment duration increases, a noticeable change in the removal of the ink spots can be observed in the figure. Applying soil and coffee to the cloth results in a noticeable stain of soil and coffee on sample (a). However, in samples (b–f), there is no noticeable spot present after being exposed to light for a duration of 6 h. Therefore, it can be inferred that the TiO_2_-coated sample, which underwent a 10 min plasma treatment, exhibited the most effective self-cleaning characteristic. The cause of this phenomenon can be attributed to the heightened stimulation of the fabric’s surface and enhanced absorption of TiO_2_. Increasing the duration of exposure to light enhances the self-cleaning ability of the sample as a result of the photocatalytic properties of TiO_2_. Additionally, the sample becomes visibly lighter. The finding is consistent with earlier studies. Haji et al. treated polyester/wool fabric with TiO_2_ nanoparticles to provide self-cleaning properties. Prior to coating the nanoparticles with TiO_2_ nanoparticles, an oxygen plasma treatment was used to promote nanoparticle loading and adhesion to the fibers. The results confirmed that the plasma treatment enhanced the number of nanoparticles loaded onto the fabric. The plasma-treated samples demonstrated better self-cleaning properties than the control samples (raw fabric and raw fabric after TiO_2_ coating).^48^ Saleem et al. discovered that plasma/TiO_2_-coated fabric had a high self-cleaning efficiency due to the firm bonding of nanoparticles on the plasma-functionalized surface.^37^ According to Bozzi et al.^49^ and Qi et al.,^50^ TiO_2_ films on polyester substrates have the ability to fully remove the color from red wine and coffee stains upon exposure to sustained light irradiation.

**Figure 10:**
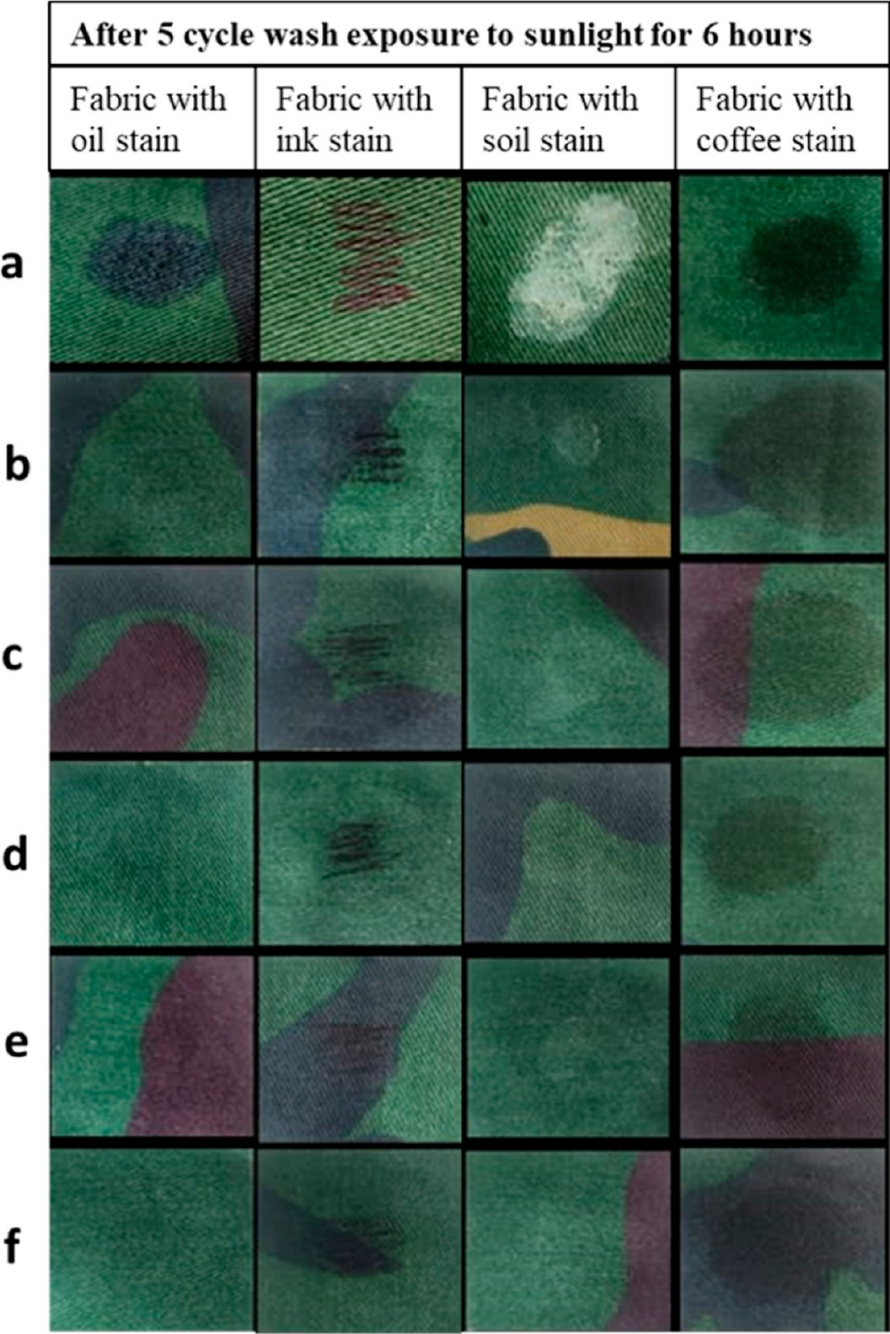
After five cycle wash exposure to sunlight for 6 h: (a) untreated sample, (b) TiO_2_-coated sample treated with plasma for 2 min, (c) TiO_2_-coated sample treated with plasma for 4 min, (d) TiO_2_-coated sample treated with plasma for 6 min, (e) TiO_2_-coated sample treated with plasma for 8 min, and (f) TiO_2_-coated sample treated with plasma for 10 min.

Figure 10 displays stained cloth that has undergone five wash cycles and has been exposed to sunshine for 6 h. The washed samples exhibited reduced self-cleaning characteristics. It is due to the removal of some TiO_2_ nanoparticles because of washing, which is a common phenomena. Regarding oil stains, samples (e) and (f) demonstrated superior self-cleaning abilities even after being washed. Nevertheless, oil stain is fading but still slight amount is visible from (b) to (d) samples which ensures that samples still have self-cleaning effect. The ink stain gradually fades from (b) to (f) once the samples are washed. Sample (f) exhibited a superior self-cleaning effect compared to the other samples for soil stains. In case of coffee stain, all the after washed samples showed a minor self-cleaning effect.

**Figure 11:**
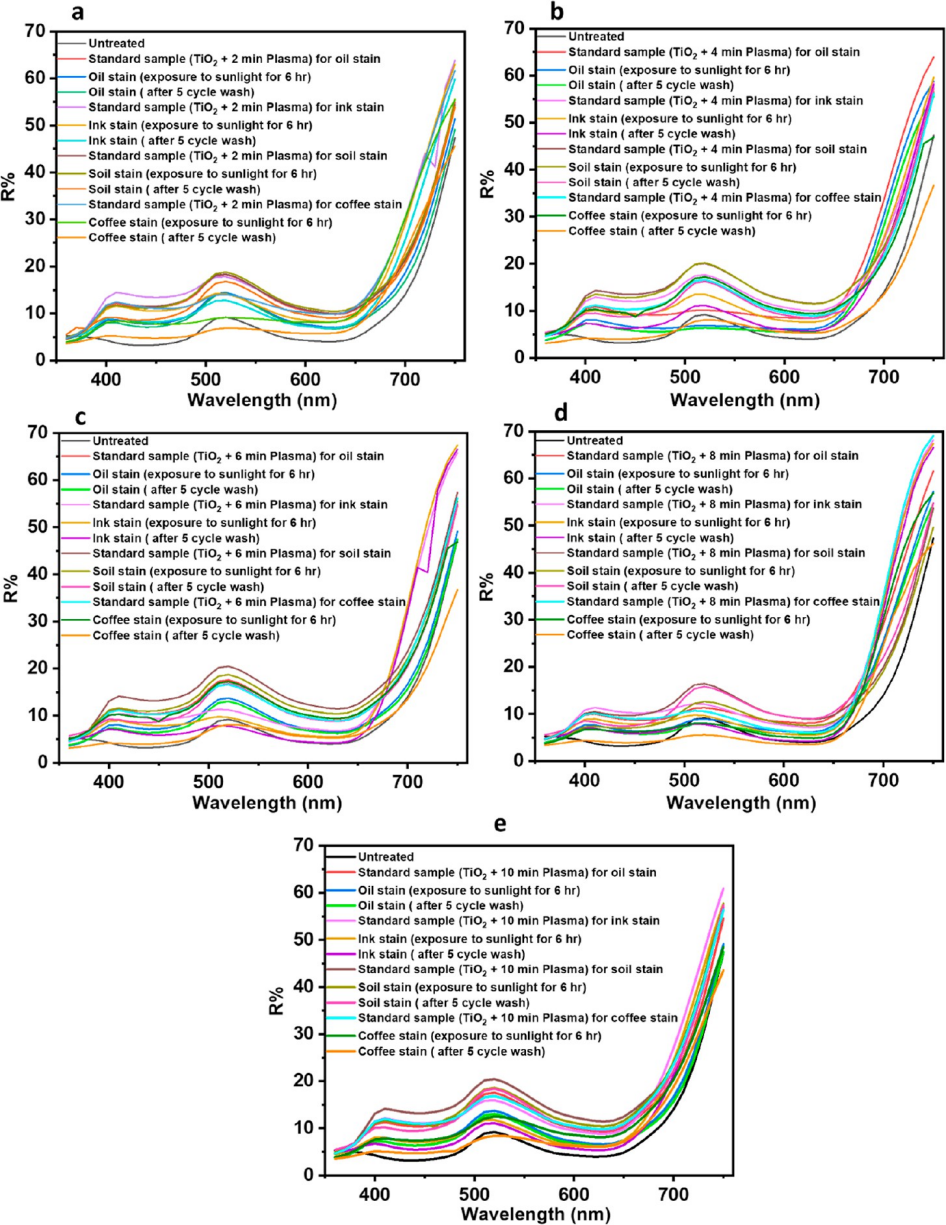
Reflection factor for oil, ink, soil, coffee stain after five washes and 6 h exposure to sunlight (a) TiO_2_-coated 2 min plasma-treated, (b) TiO_2_-coated 4 min plasma-treated, (c) TiO_2_-coated 6 min plasma-treated, (d) TiO_2_-coated 8 min plasma-treated, and (e) TiO_2_-coated 10 min plasma-treated.

The self-cleaning property has been also evaluated by measuring the reflectance value of before and after the washed samples. Figure 11 depicts the reflectance value (*R* %) obtained from the spectrophotometer. A lower reflectance value signifies a more intense darkness; thus, the untreated sample exhibits the darkest shade. When the sample is subjected to TiO_2_ coating and plasma treatment for different durations, the reflectance value reaches its maximum, and the fabric’s shade becomes the lightest for all types of stain samples. For instance, in the case of a 2 min plasma-treated oil stain sample, the reflectance values follow this sequence: TiO_2_-coated 2 min plasma-treated standard sample > exposure to 6 h sunshine > after five cycles of washing. Upon exposure to sunlight for 6 h, the reflectance value of the sample decreases as anticipated, indicating a decrease in the brightness of the shade compared to the standard sample. Nevertheless, the sample continues to exhibit a higher reflectance value compared with the untreated and washed sample, suggesting an enhancement in the self-cleaning ability as a result of the TiO_2_ photocatalyst feature. After undergoing five cycles of washing, the shade’s lightness is slightly diminished compared to exposure to a 6 h sunlight sample. However, it remains much higher than the untreated sample, indicating that the sample still possesses self-cleaning properties. The same pattern of results was observed for ink, soil, and coffee stain samples treated with TiO_2_/plasma for 4, 6, 8, and 10 min.

### 3.8 Breaking Strength

**Figure 12:**
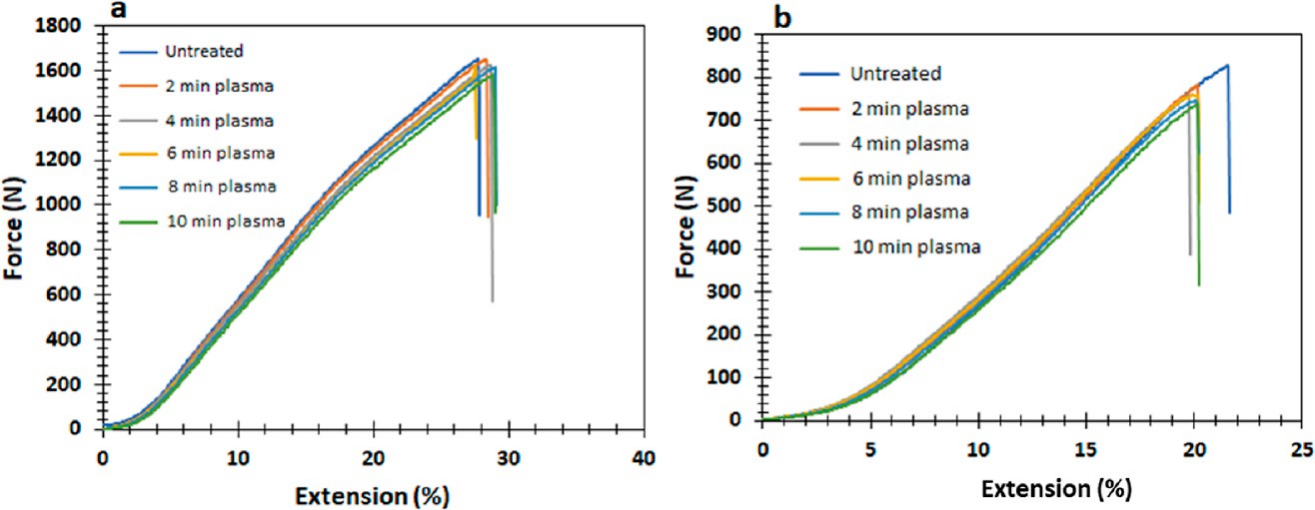
Breaking strength vs elongation graph for (a) warp direction, (b) weft direction.

Figure 12 depicts the standard force–extension graph for the untreated and plasma-treated sample, which has a nanocoating in both the warp and weft directions. The maximal breaking strength of the untreated sample is 1651 N in the warp way and 828 N in the weft way, while the minimum breaking strength of the warp direction was found to be 1581.28 N, and the least breaking strength of the weft direction was found to be 737.98 N for 10 min plasma-treated sample. The 2, 4, 6, and 8 min plasma-treated sample showed 1650.32, 1624.79, 1620.3, and 1614.28 N, respectively, in warp way and 781.40, 760.56, 758.24, and 746.98 N, respectively, in weft direction. The elongation varies from 27.88 to 28.99% as the sample trends from untreated to 10 min plasma treatment in the warp direction and from 21.63 to 20.22% in the weft direction for a similar trend. The breaking strength of the fiber is marginally diminished in both the warp and weft orientations following plasma treatment due to the weakening of chemical bonds.^51^ The breaking strength decreases very slightly with longer plasma treatment durations. Consequently, the breaking strength of the sample has not exhibited any noticeable alteration as a result of the application of plasma treatment and TiO_2_ nanocoating.

### 3.9 Color Fastness to Rubbing

**Figure 13:**
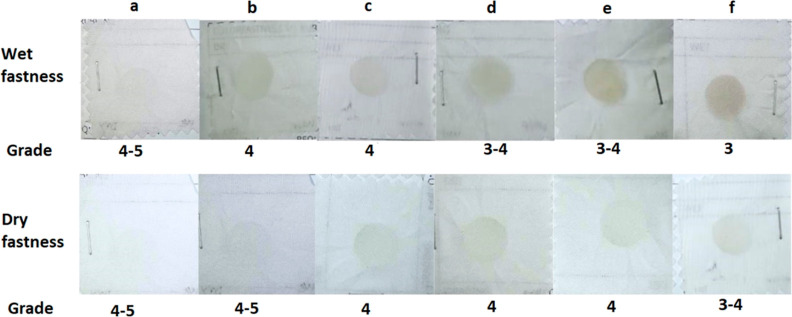
Color fastness to rubbing of (a) untreated sample, (b) TiO_2_-coated sample treated with plasma for 2 min, (c) TiO_2_-coated sample treated with plasma for 4 min, (d) TiO_2_-coated sample treated with plasma for 6 min, (e) TiO_2_-coated sample treated with plasma for 8 min, and (f) TiO_2_-coated sample treated with plasma for 10 min.

Conducting a rubbing test is essential for assessing the probability of fabric discoloration caused by friction. The color fastness to rubbing results for the wet and dry conditions are displayed in Figure 13, comparing untreated and TiO_2_-coated plasma-treated samples.

The data demonstrate that the color fastness of both wet and dry samples deteriorates with increasing duration of plasma treatment. The dry fast sample has superior fastness properties compared to those of the wet fast sample, as observed by a visual comparison between the wet and dry test samples. In the wet test, the 10 min plasma-treated sample showed a fastness rating of 3, whereas in the dry test, the identical sample showed a fastness rating of 3–4. Other authors have also documented a decrease in rubbing fastness due to plasma treatment.^52^ A modification in the interplay between the dye molecules and the fiber surface is induced by the plasma treatment.

### 3.10 Air Permeability

**Figure 14:**
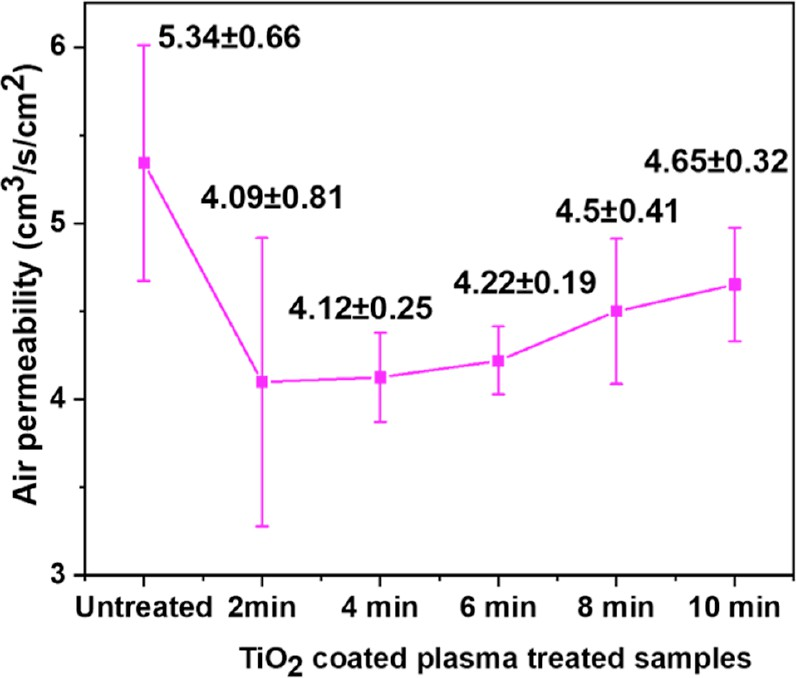
Air permeability results for untreated and TiO_2_-coated plasma-treated samples.

Cloth comfort is evaluated based on its air permeability, which is a crucial characteristic.^29^ The correlation between air permeability and plasma treatment is illustrated in Figure 14. Kale et al. found that the air permeability of a fabric decreases in proportion to the increase in the quantity of etched fiber present on its surface.^53^ The untreated sample had an air permeability of 5.34 ± 0.66 cm^3^/s/cm^2^, while the sample treated with plasma for 2 min had an air permeability of 4.09 ± 0.81 cm^3^/s/cm^2^. The air permeability demonstrates a rising trend as the duration of plasma treatment increases from 2 to 10 min. The maximum air permeability value recorded 4.65 ± 0.32 cm^3^/s/cm^2^, which is observed in the sample treated with plasma for 10 min. Based on the results obtained from EDX and SEM analysis, it can be observed that the fabric’s ability to deposit more particles on it when the plasma treatment period is extended. Nevertheless, the fabric surface displayed a greater number of etched fibers following a 2 min plasma treatment compared to a 10 min treatment. The application of nanoparticles as a surface binder helps decrease the presence of hair-like fibers on the surface. Another factor could be the alteration in the crystallinity of both cotton and polyester fibers as a result of the plasma treatment duration. Prior research also corroborates the established finding.^54^

The ANOVA results for the air permeability are shown in Table 3. Here, “*F*” represents the distribution with df (degree of freedom) for factor (untreated and plasma-treated fabrics with different time durations) and error, respectively. The significance level is denoted by the *p*-value, while *F*-value is the ratio of explained variance to total variance. The *p*-values (<0.05) for all sources indicate a statistically significant difference between the untreated and plasma-treated fabrics with various time durations.

## 4 Conclusions

This study examines the impact of TiO_2_ nanoparticle coating with various plasma treatment times on the uniform defense to improve its functional self-cleaning ability. The absorption rate of TiO_2_ on the surface of the fabric steadily increases with longer treatment duration of the air plasma, compared to the coated raw sample without plasma treatment, which is confirmed by SEM and EDX analysis. The unique characteristic of plasma treatment allows for chemical deposition in the fabric without the need for any binders. Through exposure to daylight, the various organic and inorganic contaminants such as oil, ink, soil, and coffee are readily eliminated from the fabric. Additionally, fabrics with a higher deposition of TiO_2_ clean the fabric surface more efficiently. The reason for this is the photocatalytic action of TiO_2_, which breaks down color molecules on the fabric’s surface by converting water and oxygen molecules in the air into highly oxidizing active chemicals. The stained washed sample also showed reduced self-cleaning property as confirmed by the reflectance value and visual assessment. The UVPF values exhibited a progressive increase as a consequence of the inherent UV absorption characteristics of TiO_2_ when coated with it through various plasma treatment durations, as demonstrated by the band theory of solids. The prepared sample exhibited no substantial alterations in the tensile strength, rubbing resistance, and air permeability. The ANOVA analysis of air permeability indicate a statistically significant difference between the untreated and TiO_2_-coated plasma-treated fabrics with various time durations. In conclusion, the defense uniform’s effective self-cleaning qualities can be widely utilized by defense personnel in their everyday activities, which efficiently minimizes the time required for cleaning, streamlines the maintenance process, and ensures environmental protection.

**Table 3:** ANOVA Data for Air Permeability of Untreated and TiO_2_-Coated Plasma-Treated Samples

source	df	sum of squares	mean square	*F*	*p*-value
factor/untreated and 2 min plasma-treated	1	3.86884	3.86884	6.92509	0.03011
error	8	4.46936	0.55867		
factor/untreated and 4 min plasma-treated	1	3.70881	3.70881	14.51021	0.00517
error	8	2.0448	0.2556		
factor/untreated and 6 min plasma-treated	1	3.14721	3.14721	12.99641	0.00693
error	8	1.93728	0.24216		
factor/untreated and 8 min plasma-treated	1	1.77241	1.77241	5.74227	0.04343
error	8	2.46928	0.30866		
factor/untreated and 10 min plasma-treated	1	1.37641	1.37641	5.37324	0.04907
error	8	2.04928	0.25616		
factor/untreated and all plasma-treated	5	5.56383	1.11277	4.47271	0.00507
error	24	5.97096	0.24879		
